# Correspondence

**Published:** 1870-10

**Authors:** 


### Correspondence.

Editors Register: The Register for April contains an article on Failures, in which the author says: "We recently heard a professional brother exceedingly anxious to tell all of his failures in practice. At the time it just occurred to us that if he should really do what he seemed so anxious for, he would have his hands full." In answer to which we would say, if he or any body else should succeed in doing what they should in regard to their failures, we have no doubt their hands would be full.

Again he says: "We have usually found that it is an easy matter to make a failure, almost anybody can do that." That is very true, and we will add that not only almost any body can, but every body does; and we dare say many are made that would not be if we would take a little more pains to find out why and how we make them. When we have learned to look well into our failures and see wherein we missed, we may promise ourselves a little more success. We can see no impropriety in speaking of some of our failures, especially in our society meetings, where we assemble to advise with each other. We can improve ourselves equally as much by discussing some of our failures as by talking and boasting so much over successes, that are able to take care of themselves; failures need to be cared for. This kind of seeking and investigation will put us in the right way, the bright and successful way.

Our Dental roads as a general thing are rather rough and hard to travel. They require a good deal of work to make them solid, even, and smooth, to say nothing of illumination. To travel successfully these roads, one must become something of an investigator; he must not take up all of his time in speaking of a few successes; he must investigate his own as well as others' failures. In this way we propose to get into our brother's glorious noonday light, that will enable us to open our eyes and remove the scales of ignorance.

In conclusion the article says: "Let us talk of our successes, and tell how we accomplish them, and cast off our failures with the darkness and ignorance that attaches to them."

Talk of our successes? That is very easily done; almost everybody loves to do that. But when it comes to speak of our imperfections and many miserable failures, that is a different thing. It requires courage and manliness to do that. In attempting to cast them off, we fear the attachment might take us along with them. Hence we insist upon a thorough investigation of these failures, and if we should have one of a serious character, in which we should feel our incompetence to manage, we should be delighted to have some one able and willing to assist us. We hope the writer on failures has not been bored by some one who managed to sum up courage enough to speak of a failure. It has always been our misfortune to be bored by those who never make them, so far as making them known to others is concerned.

Nashville, Sept. 15, 1870. C.

---

EDITORS REGISTER: Having received a number of communications from Dentists desiring information more than was given in the REGISTER by me in regard to aluminum plate, Starr's solder, Watt & Williams' metal, etc., etc., and not having time to answer each of these separately, I ask their indulgence and hope that they will receive what I here say as all that I could say were I to answer each individually. As to aluminum plate, you prepare the plate by swaging the same as gold or silver; it swages very easily, and you can make a very perfect fit, and with care in soldering no springing of plate will occur; but as there is a patent upon the use of Starr's solder, I can only say that you can procure a right to use it by communicating with Dr. Fairchild, of Covington, Ky., or Dr. Starr, of New York, who will give all instructions needed. Success will depend upon your efficiency as an operator. You can, by communicating with Dr. Williams, of Xenia, Ohio, get all the information in regard to Watt & Williams' metal, and their mode of mounting teeth on platinated silver plate, and the use of this metal as base plate. You ask, are not teeth mounted upon this or any of the white metals now coming into use objectionable because of the weight, they being so much heavier than rubber plates? True, the same thickness of plate is heavier, but you must recollect that the metal has so much greater strength that it is quite unnecessary to give more than one third the bulk in the piece of work, which will only give about the same weight of a good gold plate. But one says: "I am not sure of always having a perfect cast plate when it is so thin." You can be sure of your plate always by having your case properly prepared, using Geo. Watt & Co.'s flask.

I recently saw at the Dental Depot of Strong, Smith & Co., Indianapolis, a denture, the base cast of aluminum, which was very perfect indeed, and I was better pleased with its appearance than any kind of low priced work which I have seen; but I do not know enough to speak of it now; I hope to be able to do so in the future. Dr. Loomis, of Carthage, Ill., is the discoverer of this mode of mounting teeth. W. M. H.

CHICAGO, ILLS., October 3, 1870.

EDITORS DENTAL REGISTER: At the last meeting of the American Dental Association in Nashville, I noticed in the discussions relative to the healthiness of rubber plates, quite a diversity of opinion and experience.

The following case has just came under my observation, and may be of interest to some pro-rubber men, and perhaps to the profession generally. May 13, 1870, I extracted roots, etc., from the mouth of a young, strong and healthy woman, preparatory to inserting an artificial denture. August 3, inserted teeth on vulcanite or hard rubber base; mouth in as healthy condition as is usual so soon after extracting; when she left the city and did not see her again until September 3, when she again called upon me complaining of a peculiar unpleasant heating sensation under the plate, accompanied by pain in the cheeks over the region of the antrum; soreness of and pain in the eyes, with constant and copious watery discharge.

Upon removing plate found all that portion of mucus membrane covered by it, very much thickened, red, and secretions abnormal, both in quantity and quality. Upon pressing upon diseased part blood oozed from it, also superficial ulceration in at least three places; provided the patient with preparation carbolic acid and glycerin (3 grs. to ounce), and directed her to use some upon retiring at night, and two or three times daily, leaving the plate out as much as possible. Patient returned in one week, mouth much improved; took impression, and at once inserted denture upon aluminum base, which she has worn since with perfect comfort. No more pain in eyes and cheeks, and none of that "burning sensation" under the plate, and to-day, September 28, her mouth appears in as healthy condition as before she had worn a plate. I have often noticed same appearance of mucus membrane where rubber plates have been worn some time, but never have seen one so badly effected in so short a time.

That the ingredients which composed the plate were the sole cause of this trouble, I do not hesitate to assert. I do not undertake to say how. I have my own theory, and so have others.

In regard to the statements advanced that "mouths are diseased by wearing metal plates," none will deny. However, all readily remember that when only metal plates were in use, we saw nothing like the same number of diseased mouths as now, when rubber is so extensively used, and for some reason the most aggravated cases result from use of rubber plates as now worn.

Very respectfully,

E. D. SWAIN.

